# Applicable or non-applicable: investigations of clinical heterogeneity in systematic reviews

**DOI:** 10.1186/s12874-016-0121-7

**Published:** 2016-02-17

**Authors:** Laura E. Chess, Joel J. Gagnier

**Affiliations:** Department of Emergency Medicine, Henry Ford Hospital, Detroit, MI USA; Department of Orthopaedic Surgery, University of Michigan, Ann Arbor, MI USA; Epidemiology, School of Public Health, University of Michigan, Ann Arbor, MI USA

**Keywords:** Systematic reviews, Clinical heterogeneity, Meta-analyses, Cochrane Collaboration

## Abstract

**Background:**

Clinical heterogeneity can be defined as differences in participant characteristics, types or timing of outcome measurements and intervention characteristics. Clinical heterogeneity in systematic reviews has the possibility to significantly affect statistical heterogeneity leading to inaccurate conclusions and misled decision making. The aim of this study is to identify to what extent investigators are assessing clinical heterogeneity in both Cochrane and non-Cochrane systematic reviews.

**Methods:**

The most recent 100 systematic reviews from the top five journals in medicine—*JAMA*, *Archives of Internal Medicine*, *British Medical Journal*, *The Lancet*, and *PLOS Medicine—*and the 100 most recently published and/or updated systematic reviews from Cochrane were collected. Various defined items of clinical heterogeneity were extracted from the included reviews. Investigators used chi-squared tests, logarithmic modeling and linear regressions to determine if the presence of such items served as a predictor for clinical heterogeneity when comparing Cochrane to non-Cochrane reviews. Extracted variables include number of studies, number of participants, presence of quantitative synthesis, exploration of clinical heterogeneity, heterogeneous characteristics explored, basis and methods used for investigating clinical heterogeneity, plotting/visual aids, author contact, inferences from clinical heterogeneity investigation, reporting assessment, and the presence of a priori or post-hoc analysis.

**Results:**

A total of 317 systematic reviews were considered, of which 199 were in the final analysis. A total of 81 % of Cochrane reviews and 90 % of non-Cochrane reviews explored characteristics that are considered aspects of clinical heterogeneity and also described the methods they planned to use to investigate the influence of those characteristics. Only 1 % of non-Cochrane reviews and 8 % of Cochrane reviews explored the clinical characteristics they initially chose as potential for clinical heterogeneity. Very few studies mentioned clinician training, compliance, brand, co-interventions, dose route, ethnicity, prognostic markers and psychosocial variables as covariates to investigate as potentially clinically heterogeneous. Addressing aspects of clinical heterogeneity was not different between Cochrane and non-Cochrane reviews.

**Conclusions:**

The ability to quantify and compare the clinical differences of trials within a meta-analysis is crucial to determining its applicability and use in clinical practice. Despite Cochrane Collaboration emphasis on methodology, the proportion of reviews that assess clinical heterogeneity is less than those of non-Cochrane reviews. Our assessment reveals that there is room for improvement in assessing clinical heterogeneity in both Cochrane and non-Cochrane reviews.

## Background

Systematic reviews often apply statistical techniques to combine data, a meta-analysis, in order to arrive at a pooled treatment effect. However, the precision of this estimate depends on heterogeneity within and between the included studies. Clinical heterogeneity can be defined as differences in participant characteristics (e.g., age, baseline disease severity, ethnicity, comorbidities), types or timing of outcome measurements and intervention characteristics (e.g., dose, frequency of dose, training of interventionists) [1]. Clinical heterogeneity can cause significant statistical heterogeneity leading to inaccurate conclusions and ultimately misled decision making [1].

Healthcare professionals and policy makers are not commonly using systematic reviews as decision-making references despite their valuable applications [2]. Nevertheless, by teasing out factors that may influence the outcome effect, clinical heterogeneity assessment provides clinical decision makers with not only a more reliable estimate of the treatment effect, but also ideally the ability to tailor their interventions to improve the health outcomes of their patients.

Clinical heterogeneity is not to be confused with statistical heterogeneity or methodological heterogeneity. While clinical heterogeneity is the difference in intervention and outcome measurement, statistical heterogeneity is differences in results when measuring the same outcome [1]. This may include both opposite findings (benefit versus harm) or simply a difference in the extent of benefit or harm across studies. Ironically, it is possible that statistical heterogeneity could be a product of the presence of clinical or methodological heterogeneity. However, it is important to note that the lack of statistical heterogeneity should not preclude the investigation of either clinical or methodological heterogeneity.

There is no lack of resources offering reviewers routes to investigate statistical heterogeneity in systematic reviews. However, across consensus-based guidelines, statistical papers, and expert narrative reviews, there is little overlap in recommendations and only a small number of sources include a comprehensive set of recommendations regarding clinical heterogeneity [3]. Of nine methods manuals of the organizations or public-sector agencies that produce the largest number of systematic reviews, only five— U.S. Agency for Healthcare Research and Quality, Evidence-based Practice Centers Program (AHRQ EPC Program); Centre for Reviews and Dissemination (CRD); Cochrane Collaboration; Oregon Health & Science University Drug Effectiveness Review Project (DERP); European Network for Health Technology Assessment (EUnetHTA)—provided definitions of clinical heterogeneity, of which Cochrane provides the most detailed discussion [4]. Cochrane Handbook defines the three types of heterogeneity, explains how they differ, and offers advice regarding forest plots, a priori subgroup analysis and meta-regression to assess clinical heterogeneity [1]. Thus, the aim of this study is to identify to what extent investigators are assessing clinical heterogeneity in both Cochrane and non-Cochrane systematic reviews.

## Methods

### Study Selection

The most recent 100 systematic reviews published in 2012 were collected from five of the top journals in medicine as determined by impact factors as listed in the 2011 Thompson ISI citation reports of General and Internal Medicine: *Journal of American Medical Association* (JAMA), *Archives of Internal Medicine* (AIM), *British Medical Journal* (BMJ), *The Lancet*, and *PLOS Medicine*. To note, due to its limited number of annual systematic review publications, *New England Journal of Medicine* (NEJM) was not included in this study despite its high impact factor. Additionally, investigators retrieved the 100 most recently modified systematic reviews from the Cochrane Database published in 2012. A total of 317 of the most recently updated/published papers were initially considered for inclusion, however studies were excluded because they were only updated abstracts, author name changes or shortened versions of previously published reviews. Papers included were reported as being systematic reviews or meta-analyses only from any field of medicine.

### Data Extraction

Investigators extracted data relating to number of studies included, total number of participants (i.e., the sample size across all included studies in each review), expertise of the review team members (by referring to their degrees/training and to relevant information on web pages at their respective institutions), presence of quantitative synthesis, exploration of clinical heterogeneity, clinically heterogeneous characteristics explored, basis for exploring clinical heterogeneity, methods used in investigating clinical heterogeneity, plotting and visual aids, author contact, inferences from clinical heterogeneity investigation, reporting assessment, and a priori vs. post-hoc analysis (Table 1) [5]. All data were extracted by one individual (LC) and cross-checked by an experienced methodologist and physician (JG).

**Table 1:** Criteria for investigating clinical heterogeneity in systematic reviews [5]

Review team	It is recommended to have at least one or two individuals with clinical expertise, and at least one or two individuals with methodological expertise in systematic reviews/meta-analyses and on the type of study designs that are included. The team should recognize their own biases and attempt to compensate by including members with a wide range of (potentially conflicting) beliefs.
Quantitative synthesis	Did the review perform a quantitative synthesis?
Clinical heterogeneity variables	Did the authors choose characteristics for exploring that can be considered aspects of "clinical heterogeneity"? Patient level: Age, baseline disease severity, sex, gender, ethnicity, comorbidities, genetic, other psychosocial variables, and other important features of the disease. Intervention level: Dose/strength/intensity of treatment, duration of treatment, brand/manufacturer, cointerventions, timing, route of administration, compliance, clinician training, implementation, other. Outcome level: Event type, outcome measure type, outcome definition, length of follow-up, timing of outcome measurement(s). Other: Research setting, geographical issues, length of follow-up.
Planned clinical heterogeneity exploration	Did the authors describe how they planned to investigate differences between studies? All investigations of clinical heterogeneity should ideally be pre-planned a priori and not be driven by observing the data. But methods for looking at data to identify unanticipated variables of interest (i.e., post-hoc investigations) need to be pre-specified as well (e.g., looking at summary tables, graphical displays). Describe the following: which variables you will investigate, how this will be done, when you will perform these investigations, and how results will be interpreted and incorporated into your results and conclusions.
A priori vs. post-hoc	Was it a priori or post-hoc? Reviewers should think through all potentially relevant variables to explore and not rely on statistical measures of heterogeneity to justify such investigations.
Individual patient data (IPD) vs. aggregate patient data (APD)	Was IPD, APD or a combination used? APD = Summary or aggregate data from trials only. This is subject to ecological bias, that is, investigations of trial-level variables are valid (e.g., dose, duration), while investigations of patient-level variables are not (e.g., age, baseline severity). IPD = Original individual data on each patient. This type of data is valid for both trial-level and patient-level variables. But, one must control for baseline difference between the patients across trials. Consider contacting authors and reviewing protocols of primary studies where available. Obtaining IPD for investigating clinically related patient-level variables is ideal.
Parsimony—number of investigations to perform and variables to explore	Was parsimony used in choosing variables to explore? Use parsimony as a guide to such investigations. A rule of thumb for the number of trials is that there should be close to ten trials when working with summary or aggregate patient data (APD) or ten individuals per variable, when working with pooled or individual patient data (IPD). Consider making a hierarchy of clinically related variables and investigate only those variables for which your rationale and power are sufficient.
Clinical heterogeneity variables not later investigated	Were there characteristics that were chosen that were not eventually investigated? Variables that were mentioned to potentially contribute to clinical heterogeneity which were ultimately never investigated (or reported) in the analysis.
Outliers/sensitivity analysis	When there are individual trials that are clear outliers, was there an attempt to determine why? (e.g., was a sensitivity analysis done, where these trials are eliminated and effect estimate changes? When there are individual trials that are clear outliers, attempt to determine why and consider a sensitivity analysis to eliminate these trials and observe how the effect estimate changes. One may also consider an influence analysis, in which the effect of deleting individual studies from the analysis on the overall estimate is explored.
Statistical heterogeneity	Was statistical heterogeneity assessed?
Statistical heterogeneity as prerequisite to investigate clinical heterogeneity	Was statistical heterogeneity used as a prerequisite for investigating clinical variables? Reviewers should think through all potentially relevant variables to explore and not rely on statistical measures of heterogeneity to justify such investigations. Clinical heterogeneity related to specific individual factors could be present even in the absence of a significant statistical test for the presence of heterogeneity (e.g., Cochran’s Q test)
Plots/visuals	Were plotting or other visual aids used to explore reasons for clinical heterogeneity? Consider using graphical displays of data from trials to help identify potential clinical reasons for heterogeneity. Examples of plotting and visual aids of the data include: summary data sheets, forest plots, L’Abbé plots, funnel plots, Galbraith plots/radial plots, influence plots, dose/response curves, multidimensional scaling, and heat maps.
Cautious inferences	Was caution used in making inferences from the findings of investigations of heterogeneity? Results are generally observational and thus hypothesis generating only. Authors should express the validity of and confidence in their findings. When interpreting results of these investigations it is suggested to consider: confounding, other sources of bias (e.g., publication, misclassification, dilution, selection), magnitude and direction of effect and CI, and thinking through the plausibility of causal relationships. It may not be appropriate to conclude that there is consistency of effect if subgroup effects are not found [20]. Authors should use their findings to make specific recommendations about how future research could proceed or build upon these results (not just conclude that “more research is needed”).
Sufficient reporting	When there was insufficient information, were the study authors contacted for more information?
Reporting at a limitation	Was the reporting in the included studies assessed and commented on as a potential problem for investigating clinical heterogeneity? Consider the potential for lack of reporting of data or information relating to clinical variables in the primary studies. Consider contacting the authors for missing or additional data on important clinical variables. Reviewers must be careful to report all of their proposed and actual investigations of clinical heterogeneity. The PRISMA statement should be adhered to when reporting their reviews.

### Statistical Analyses

Investigators conducted a chi-squared comparison of Cochrane versus non-Cochrane studies regarding number of studies included, total number of participants, presence of quantitative synthesis, exploration of clinical heterogeneity, clinically heterogeneous characteristics explored, basis for exploring clinical heterogeneity, methods used in investigating clinical heterogeneity, plotting and visual aids, author contact, inferences from clinical heterogeneity investigation, reporting assessment, and a priori vs. post-hoc analysis (Table 2). Investigators performed logistic regression with Cochrane or non-Cochrane systematic reviews being the response variable and the aforementioned components of clinical heterogeneity investigations being the predictors (Table 3) followed by a deletion process (Table 4).

**Table 2:** Chi-squared analysis of Cochrane status by individual item scores

	N, % = yes (Cochrane)	N, % = yes (Non-Cochrane)	*p* value
Review team	1 (1.0)	5 (5.1)	0.091
Quantitative synthesis	63 (63.0)	81 (81.8)	**0.0030**
CH variables	90 (90.0)	80 (80.8)	0.066
Planned CH exploration	89 (89.0)	73 (73.7)	**0.0057**
A priori vs. post-hoc	11 (11.0)	34 (34.3)	**<0.0001**
Parsimony	65 (65.0)	93 (93.9)	**<0.0001**
CH not investigated	56 (56.0)	29 (29.3)	**0.0001**
Outliers/sensitivity	40 (40.0)	50 (50.5)	0.14
Proper reporting	84 (84.0)	42 (42.4)	**<0.0001**
Statistical hetero-geneity (SH)	74 (74.0)	85 (85.9)	**0.037**
SH used for investigating CH	2 (2.0)	2 (2.0)	0.38
Plots/visual aids	20 (20.0)	11 (11.1)	0.084
Cautious inferences	68 (68.0)	86 (86.9)	**0.0015**
Reporting as limitation	30 (30.0)	22 (22.2)	0.20

*CH* clinical heterogeneity

Bolded *p* value indicates significance

**Table 3:** Logistic regression for being a Cochrane Review (dependent/response variable) by various predictor variables

	OR	95 % CI	*p* value
Number of studies	0.94	0.92–0.97	**<0.0001**
Number of participants (log)	0.14	0.077–0.24	**<0.0001**
Quantitative synthesis (1 = yes, 0 = no)	0.38	0.20–0.73	**0.0035**
Individual patient data (IPD; 1 = yes, 0 = no)*	n/a		n/a
Aggregate patient data (APD; 1 = yes, 0 = no)	0.28	0.15–0.53	**0.0001**
Combination of IPD & APD (1 = yes, 0 = no)	1.00	0.24–4.11	1.00
Investigated an aspect of clinical heterogeneity (1 = yes, 0 = no)	2.14	0.94–4.87	0.070

Note: 1. Bolded *p* value indicates significance. 2. OR of greater than 1 favors being a Cochrane Review for each variable

*No Cochrane studies conducted individual patient data analysis

**Table 4:** Logistic regression with stepwise deletion for being a Cochrane Review (dependent/response variable) by various predictor variables

	Model 1 (OR, *p* value)	Model 2 (OR, *p* value)
Number of studies	0.96, **0.0011**	0.96, **0.0015**
Number of participants	1.00, **0.025**	1.00, **0.025**
Quantitative synthesis (1 = yes, 0 = no)	0.087, **0.0014**	0.12, **0.0006**
APD 1 = yes, 0 = no)	1.48, 0.51	
Combination of IPD and APD (1 = yes, 0 = no)	12.9, **0.054**	8.72, 0.066
Investigated an aspect of clinical heterogeneity (1 = yes, 0 = no)	5.29, **0.016**	5.08, **0.019**

Note: 1. Bolded *p* value indicates significance. 2. OR of greater than 1 favors being a Cochrane Review

## Results

**Fig. 1:**
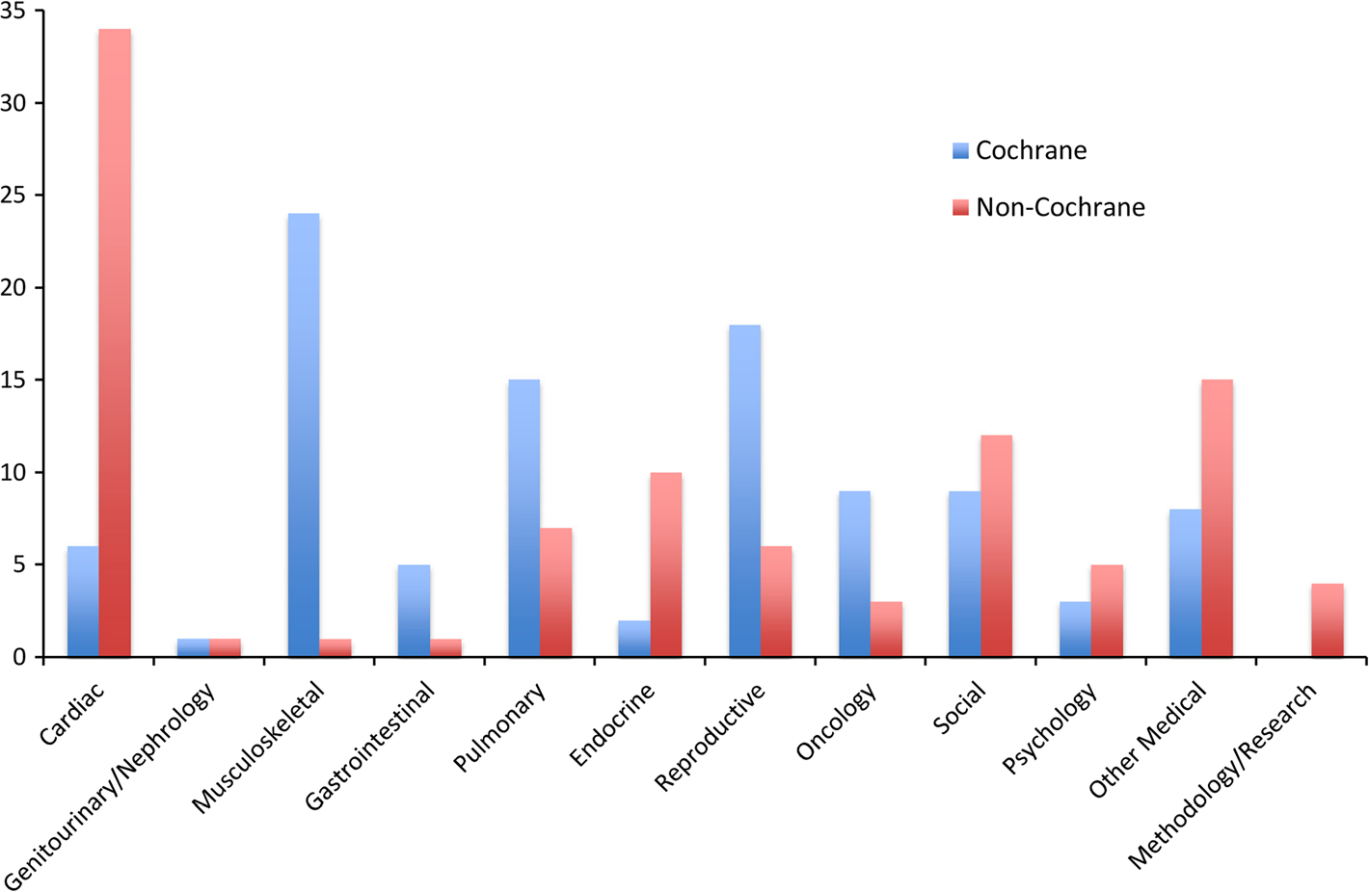
Selected Cochrane and non-Cochrane studies arranged by discipline

A total of 317 systematic reviews were considered, of which 199 were in the final analysis. The topics of Cochrane and non-Cochrane reviews differed with questions related to musculoskeletal, pulmonary and reproductive areas and non-Cochrane reviews more often relating to cardiac, endocrine and other medical conditions (see Fig. 1). Overall, the assessment and measurement of clinical heterogeneity varied greatly. A total of 81 % of Cochrane reviews and 90 % of non-Cochrane reviews explored characteristics that are considered aspects of clinical heterogeneity and also described the methods they planned to use to investigate the influence of those characteristics. The most commonly mentioned variables were age, sex, comorbidities, setting, geographic location, severity of disease and dose/dosing frequency. Unfortunately, only 1 % of non-Cochrane reviews and 8 % of Cochrane reviews explored all those clinical characteristics they initially chose. Additionally, very few studies mentioned clinician training, compliance, brand, co-interventions, dose route, ethnicity, prognostic markers and psychosocial variables as co-variates to investigate as potentially clinically heterogeneous.

In regards to comprehensive heterogeneity assessment, several measured areas of analysis in both Cochrane and non-Cochrane reviews were lacking. Only 49 % of non-Cochrane and 40 % of Cochrane reviews performed a sensitivity analysis to assess for outliers. Cochrane reviews (83 %) were however much better than non-Cochrane reviews (41 %) at contacting authors regarding missing data.

In forming and describing an appropriate team for sufficient planning and analysis, only 5 % and 2 % of all systematic reviews reported having researchers with methodological expertise in non-Cochrane and Cochrane reviews, respectively. Regarding data analysis, 81 % of non-Cochrane reviews included a meta-analysis compared to 62 % of Cochrane reviews. The majority of reviews made general suggestions regarding the importance of aspects relating to clinical heterogeneity but did not discuss the impact of these aspects on their analysis.

Another important aspect to assessing clinical heterogeneity is the transparency and truthful reporting of heterogeneity assessment and inferences. Arguably, limited reporting was a chief impediment to assessing the clinical heterogeneity analysis of these systematic reviews. However, only 22 % of non-Cochrane and 30 % of Cochrane reviews acknowledged reporting as a problem for investigating clinical heterogeneity.

A chi-squared test of the proportional percentage of “yes” for item scores showed that authors were more likely to describe how they planned to investigate clinical heterogeneity in Cochrane reviews, even though they were less likely to have performed a quantitative synthesis (Table 2). Additionally, chi-squared analysis revealed statistical favoritism towards non-Cochrane studies in performing a parsimonious and a priori analysis, as well as assessing statistical heterogeneity. However, Cochrane reviews also had proportionally increased contact with study authors if there was insufficient reporting. Lastly, a greater proportion of non-Cochrane reviews showed caution in making inferences from the findings of investigations of heterogeneity while Cochrane reviews were more likely to have characteristics that were chosen but not eventually investigated (Table 2).

From the logistic regression modeling, when comparing Cochrane and non-Cochrane studies, quantitative synthesis (OR = 0.38, CI = 0.20–0.73), greater number of studies (OR = 0.94, CI = 0.92–0.97) and aggregate patient data (OR = 0.28, CI = 0.15–0.53) were more likely to be included in the non-Cochrane systematic review (Table 3). Addressing aspects of clinical heterogeneity was not a significant predictor of whether the study was of Cochrane or non-Cochrane origin (OR = 2.14, CI = 0.94-4.87, *p* = 0.070).

## Discussion

Inevitably, studies in systematic reviews will differ to varying extents. How these differences are identified and assessed influences the overall value of a systematic review. Our assessment reveals that there is room for improvement in assessing clinical heterogeneity in both Cochrane and non-Cochrane reviews. Despite Cochrane Collaboration emphasis on methodology, the proportion of reviews that assess clinical heterogeneity is less than those of non-Cochrane reviews. It is suggested that reviewers would benefit from a clear set of universal guidelines as to how to assess clinical heterogeneity.

More problematic is that clinical heterogeneity is difficult to measure because of a lack of reporting, poorly described interventions and incomplete details of participant characteristics. One of the strengths of this study is its search of recently published reviews from across various medical arenas. This may also mean a difference in journal reviewer emphasis on reporting clinical heterogeneity. However, it is important to note that this study included the 100 most recently published systematic reviews in selected journals and is largely not a comprehensive search of all reviews across all areas of medicine. Several of the reviews we investigated did not have enough information to properly determine if and how well they assessed clinical heterogeneity. While reporting is a common problem, there are no universal guidelines on assessing clinical heterogeneity. This lack of education is ultimately impeding the ability to accurately determine a review’s generalizability and could result in drastically skewed treatment effect estimates. It is important to note that a lack of complete reporting does not necessarily imply poor assessment. Thus in addition to investigating the methods of clinical heterogeneity, it is important that authors and reviewers ensure the clear and complete reporting of such assessments.

There are several potential limitations to our study. First, we included only 200 systematic reviews that were published in 2012. Therefore, our results may not generalize to other years, to other journals or to other Cochrane reviews. Second, data were extracted by one individual and cross-checked by an experienced statistician and physician. Ideally, all data would be independently extracted by two reviewers and compared to avoid bias. Next, we did not account for details of the instructions to authors in these journals. That is, the journals included in our study may have variable reporting or method guidelines for systematic reviews (e.g., PRISMA guidelines) [6]. Thus, published reviews in these journals will be influenced by such guidance, resulting in different assessments of clinical heterogeneity and ultimately of these reviews. Future research in this area could review the instructions to authors to determine their potential influence on investigations of clinical heterogeneity.

Measuring clinical heterogeneity requires some planning. We recommend investigators pre-plan what variables they want to investigate and discuss why they chose those variables. This may entail soliciting experts in the discipline to recommend what variables they feel are important to clinical decision-making. This might include inviting coinvestigators who have clinical or content expertise on the specific question or topic of the review. Additionally, investigators should decide which method to use to determine the effects of those variables (i.e., plots, contacting authors, statistical procedures, etc.). It is important to decide upon these criteria before data analysis and state in the meta-analysis that they did so. The planning team should consist ideally of at least two individuals—one with clinical expertise and one with expertise in systematic reviews or meta-analyses. As before, this team should be acknowledged, described, and critiqued in the methods section of the review.

In addition, we recommend investigators perform a post-hoc analysis. This may include looking at summary data sheets, looking at forest plots from meta-analyses and/or utilizing L’Abbe plots, dose–response curves, funnel plots, Galbraith plots and influence plots. After identifying all the variables of interest (both pre-planned and post-hoc), authors should have ideally at least 10 trials per variable to ensure power [7, 8]. To explore these variables, investigators should employ the use of subgroup analyses and/or meta-regressions. This should be followed up with sensitivity analyses to test the robustness of findings relative to decisions made in the review process. The method and reporting of investigating clinical heterogeneity should be transparent and propose future investigations to improve on implemented methods. In particular, we recommend that systematic reviews closely follow reporting guidelines for systematic reviews (PRISMA) [6] and recent guidance for investigating clinical heterogeneity [5].

## Conclusions

In summary, defining the clinical characteristics that influence the intervention helps to tease out what population would most benefit from the intervention, or conversely who would be most harmed by that intervention. It is advised that all systematic reviews assess clinical variables and consider potential sources of heterogeneity via analysis, visual aids and with the help of individuals with methodological expertise in systematic reviews.

## Abbreviations

AHRQ EPC Program: U.S. Agency for Healthcare Research and Quality, Evidence-based Practice Centers Program
CRD: Centre for Reviews and Dissemination
DERP: Oregon Health & Science University Drug Effectiveness Review Project
EUnetHTA: European Network for Health Technology Assessment

## References

[1] Higgins JPT, Green S (eds): Cochrane Handbook for Systematic Reviews of Interventions. Version 5.0.1 [updated September 2008]. The Cochrane Collaboration, 2008. Accessed at: www.cochrane-handbook.org.

[2] Laupacis A, Straus S (2007). Systematic reviews: Time to address clinical and policy relevance as well as methodological rigor. Ann Int Med.

[3] Gagnier JJ, Moher D, Boon H, Beyene J, Bombardier C (2012). Investigating clinical heterogeneity in systematic reviews: a methodologic review of guidance in the literature. BMC Med Res Methodol.

[4] West SL, Gartlehner G, Mansfield AJ, Poole C, Tant E, Lenfestey N, Lux LJ, Amoozegar J, Morton SC, Carey TC, Viswanathan M, Lohr KN: Comparative Effectiveness Review Methods: Clinical Heterogeneity [Internet]. Rockville, MD: Agency for Healthcare Research and Quality (US); 2010 Sep. 3, Results. Available from: http://www.ncbi.nlm.nih.gov/books/NBK53317/21433337

[5] Gagnier JJ, Morgenstern H, Altman DG, Berlin J, Chang S, McCulloch P (2013). Consensus-based recommendations for investigating clinical heterogeneity in systematic reviews. BMC Med Res Methodol.

[6] Moher D, Liberati A, Tetzlaff J, Altman DG (2009). The PRISMA Group: Preferred reporting items for systematic reviews and meta-analyses: the PRISMA statement. PLoS Med.

[7] Higgins JPT, Green S (eds): Cochrane Handbook for Systematic Reviews of Interventions. Version 5.1.0 [updated March 2011]. The Cochrane Collaboration, 2011. Accessed at: www.cochrane-handbook.org.

[8] Altman DG (1991). Practical statistics for medical research.

